# Mortality in Tonga over three triennia, 2010–2018

**DOI:** 10.1186/s12889-020-10023-w

**Published:** 2021-01-06

**Authors:** Carah Figueroa, Christine Linhart, Latu Fusimalohi, Sioape Kupu, Gloria Mathenge, Stephen Morrell, Richard Taylor

**Affiliations:** 1 School of Population Health, University of New South Wales, UNSW Sydney, Sydney, Australia; 2 Ministry of Health, Nuku’alofa, Tonga; 3 Statistics for Development Division, Pacific Community, Nouméa, New Caledonia

**Keywords:** Tonga, Mortality, Infant mortality, Under-5 mortality, Adult mortality, Life expectancy

## Abstract

**Background:**

Tonga is a South Pacific Island country with a population of 100,651 (2016 Census). This study examines Tongan infant mortality rates (IMR), under-five mortality rates (U5MR), adult mortality and life expectancy (LE) at birth from 2010 to 2018 using a recent collation of empirical mortality data over the past decade for comparison with other previously published mortality estimates.

**Methods:**

Routinely collected mortality data for 2010–2018 from the Ministry of Health, national (Vaiola) hospital, community nursing reports, and the Civil Registry, were consolidated by deterministic and probabilistic linkage of individual death records. Completeness of empirical mortality reporting was assessed by capture-recapture analysis. The reconciled data were aggregated into triennia to reduce stochastic variation, and used to estimate IMR and U5MR (per 1000 live births), adult mortality (15–59, 15–34, 35–59, and 15–64 years), and LE at birth, employing the hypothetical cohort method (with statistical testing). Mortality trends and differences were assessed by Poisson regression. Mortality findings were compared with published national and international agency estimates.

**Results:**

Over the three triennia in 2010–2018, levels varied minimally for IMR (12–14) and U5MR (15–19) per 1000 births (both ns, *p* > 0.05), and also for male LE at birth of 64–65 years, and female LE at birth 69–70 years. Cumulated risks of adult mortality were significantly higher in men than women; period mortality increases in 15–59-year women from 18 to 21% were significant (*p* < 0.05). Estimated completeness of the reconciled data was > 95%. International agencies reported generally comparable estimates of IMR and U5MR, with varying uncertainty intervals; but they reported significantly lower adult mortality and higher LE than the empirical estimates from this study.

**Conclusions:**

Life expectancy in Tonga over 2010–2018 has remained relatively low and static, with low IMR and U5MR, indicating the substantial impact from premature adult mortality. This analysis of empirical data (> 95% complete) indicates lower LE and higher premature adult mortality than previously reported by international agencies using indirect and modelled methods. Continued integration of mortality recording and data systems in Tonga is important for improving the completeness and accuracy of mortality estimation for local health monitoring and planning.

**Supplementary Information:**

The online version contains supplementary material available at 10.1186/s12889-020-10023-w.

## Background

Trends in mortality are a key indicator of population health and the functioning and needs of health systems. Accurate mortality data is required for monitoring progress towards the Sustainable Development Goal (SDG) targets for reducing under-five mortality (SDG 3.2) and premature cause-specific mortality (SDGs 3.4, 3.6, 3.D, 3.9, and 16.1), and improving data availability and statistical capacity (SDGs 17.18 and 17.19) [1]. Accurate estimates of mortality by age, sex, and period are critical for planning and evaluating health policies and programmes particularly in resource-constrained countries. However, only 40% of deaths worldwide are registered, and the availability and quality of mortality data varies substantially [2] particularly across the Pacific island region [3].

Small island developing states, including 20 in the Pacific Island region, have population sizes of less than one million [4]. They are diverse in their geography, cultures, economies, and health systems, and experience challenges in obtaining accurate and timely mortality statistics because of widely dispersed populations, limited technical and financial resources, inconsistent implementation of civil registration legislation and systems, and inadequate use of existing vital registration data and other administrative records [5, 6]. Nevertheless, there have been improvements to mortality reporting systems across the region in the past two decades [2, 7, 8].

Published mortality estimates derived using indirect methods and model life tables from Census analyses have been shown to underestimate mortality and consequently overestimate life expectancy (LE) in Pacific Island countries, especially in circumstances where there are indications of considerable premature adult mortality due to non-communicable diseases (NCD) [Tonga: [9, 10]; Fiji: [11, 12]; Kiribati: [13]]. Mortality indices produced by international agencies are often based on incomplete vital registration data, model life tables from previous periods and different populations, assumptions of an expected improvement each year; or projections from previous trends, and have produced questionable and improbable estimates [3, 9, 10]. Inadequate empirical data to estimate mortality indicators hampers locally informed policy formulation and priority setting. Collaborative research studies in small countries involving local government agencies, regional/international agencies and universities make it possible to analyse and critically assess all available data sources [3, 14].

Tonga is a South Pacific Polynesian Island nation whose 2016 national Census population was 100,651, of which 39% were aged 15 years or younger, and 9% 60 years and older [15]. Tonga comprises 36 inhabited islands across five major groups; over 70% of the population lives on the largest island, Tongatapu. Tonga’s health services are provided through a national hospital (Vaiola), three outer island hospitals, 14 community health centres, and 34 reproductive, maternal and child health clinics [16].

**Fig. 1:**
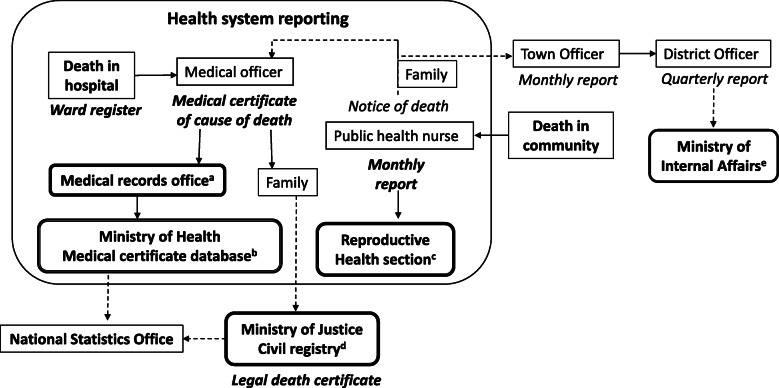
Routine death reporting processes in Tonga. Dashed lines denote gaps in reporting. ^a^ Hospital discharge records are updated in the main hospital (Vaiola) information system. The causes of death on medical certificates of cause of death (MCCD) are coded using the International Classification of Diseases 10th revision (4th edition). ^b^ Medical certificates of cause of death (MCCD) are entered into the mortality database at the Health Planning and Information division of the Ministry of Health. ^c^ Community nursing reports are collated. ^d^ Legal death certificates are entered into the registry database. ^e^ Town and District Officer reports were not available for analysis

The routine death reporting systems that operate in Tonga are: (1) the mortality database based on medical certificates of cause of death (MCCD) managed by the Ministry of Health (MoH) Health Planning and Information Division; (2) the national hospital (Vaiola) information system records of deceased at hospital discharge (separation); (3) Reproductive Health Services (RHS) data from community nursing reports; (4) The Civil Registry of Births, Deaths and Marriages managed by the Ministry of Justice; and (5) Town and District Officer reporting to the Ministry of Internal Affairs (not accessed in this analysis) (Fig. 1). MCCD are issued for deaths occurring in health facilities. For deaths that occur at home or in the community, in the absence of an authorised certifier, a notice of death form is completed by public health nurses at community health centres. Notice of death forms are also recorded in monthly community nursing reports, which are collated at the Reproductive Health Section of the hospital (Vaiola). Locally elected Town Officers are required to verify notice of death forms before a MCCD is completed [17]. In practice, a MCCD is only completed for a notice of death form at the request of the family of the deceased. Data on all MCCD issued in Tonga are entered into the MoH MCCD database.

The registration of births and deaths is required by law [18]. A MCCD is generally requested to complete the death registration, but it is not legally required and does not always occur. Registered death records at local registries are forwarded to the national civil registry office. Town and District Officers are required to submit monthly reports of deaths reported within their area to the Ministry of Internal Affairs for electoral roll purposes [19].

The routine death reporting systems collectively cover the whole country; however, some deaths may not be recorded (or captured) in any system, while others may be recorded across multiple systems. Reconciling data from multiple sources improves the validity of the derived mortality estimates, and capture-recapture assessments can be used to estimate the reporting completeness of the reconciled data. Previous manual reconciliation of available medical certification data, Civil Registry data, and community nursing reports by Hufanga et al. [9] and Carter et al. [5], estimated the reconciled data for 2005–2009 as 58–88% complete.

Within the past decade, the MoH introduced policies and procedures to improve death reporting, certification and registration in Tonga. These include enhancing the role of medical records staff to ensure the completion of death certificates by clinicians [20], and requiring the completion of death certificates for hospital deaths and notification of community deaths within 24 h of death [5, 17]. MoH policy requires all reported deaths to be medically certified. The Ministry of Justice has also improved the management of Civil Registry records [21]. Routine validation of the MoH birth and mortality datasets against the Civil Registry data [5, 21] has been implemented. Work by the authors (CF, CL, LF) has also contributed to improving the completeness and accuracy of records in the MoH MCCD database and the RHS collection. Given the increased availability of these data, it is now possible to produce nationally representative mortality estimates based on updated and reconciled deaths and denominators.

The present study investigates levels of all-cause infant, under-five and adult mortality, and LE at birth in Tonga by sex over 2010–2018. Empirical mortality data sources were reconciled and the completeness of death reporting in these systems was assessed. Published estimates by national and international agencies were also assessed and compared with the empirical results.

## Methods

This study: (1) assesses the completeness of empirical death collections using person-based linkage of multiple data sources, and capture-recapture analyses for 2010–2018; (2) calculates empirical levels of all-cause infant, under-five and adult mortality, and LE at birth, by sex over 2010–2018 based on the reconciled national death data and denominators; and (3) compares the 2010–2018 empirical results to estimates by international agencies and previous published reports.

### Analyses of 2010–2018 empirical Tonga mortality data

#### Data collection

Mortality data for 2010–2018 were obtained from four sources: the MoH MCCD database, the Vaiola hospital information system (‘deceased’ discharge records), RHS section (community nursing reports), and the Civil Registry database. Town and District Officer reports from the Ministry of Internal Affairs were not available for analysis. Hospital discharge records of deaths were included to ensure all hospital deaths were captured. Not all deaths that have an issued MCCD (and subsequently recorded by the civil registry) may be recorded in the MoH MCCD database, as manual transfer procedures within the hospital to the MCCD database are not necessarily complete. Overseas deaths and deaths among non-residents and foreign nationals were excluded in order to focus on Tongan deaths, and minimise the possibility of non-residents being included in the analysis. The deaths of Tongan citizens overseas may be registered at the civil registry by close family members of the deceased. Tongan citizens may have been overseas for a short period to receive medical treatment, or living abroad for an extended period, prior to death; as data on this period is unavailable, all overseas deaths have been excluded.

As denominators for the calculation of infant mortality rate (IMR) and under-five mortality rate (U5MR), live births in 2010–2018 were obtained from the MoH live birth database (data from certificates of live births), and the Civil Registry database, which are considered > 95% complete when compared against direct and indirect estimates of births from the census [22], and estimates from the Pacific Community calculated from crude birth rates and population estimates [4, 23]. The MoH birth database includes both hospital births and births notified in community health centres. The certificate of live birth from the MoH is primarily used for birth registration. Overseas births were excluded.

#### Deduplication and reconciliation of births and deaths

Duplicate death records were removed from the individual death data sources: 35 records in the civil registry, 107 in the medical certificate database, 118 in RHS reports, but none from the hospital information system. The death datasets were then combined, and duplicate records between each source (within the combined dataset) were linked and deduplicated. The birth datasets were similarly reconciled. Deterministic and probabilistic record linkage was performed, employing a SAS application, The Link King v9.0 [24].

Deterministic linkage uses specific criteria with varying levels of agreement among several individual identifying variables, including names, age, date of death and address between records and between data sources. Multiple criteria are used to link records at different certainty levels. For example, criteria at the highest certainty level are that first, middle and last names are exact matches, or spelling and phonetics closely match, and the dates of death match. At moderately high certainty, the names or date elements match exactly, or two of the three name or date elements match positionally.

Probabilistic linkage uses likelihood scores for the degree of similarity between each identifying variable between records. The scores are calculated from agreement and disagreement weights calculated for each variable that reflect how strong a variable is in determining duplicates; for example, an agreement on name is stronger than agreement on gender. Records are also linked at different certainty levels based on their probabilistic scores. The deterministic criteria and probabilistic linkage protocols are described in detail elsewhere [25, 26].

The matching variables used to link the death datasets were: name (first, middle, last, mother’s name); sex; age; date of death; and island group of death. Date of birth and place of residence are collected in some, but not all death datasets, and thus were used alternatively in place of the age variable in multiple rounds of deduplication. The matching variables for the birth datasets were: names (first, middle, last), mother’s name; father’s name; sex; date of birth; place of birth, and island group of birth. Datasets were pre-processed to standardise the format of the matching variables. Any records with unknown sex (*n* = 28) or age (*n* = 100) were proportionately redistributed among the remaining dataset, first by sex, then by age. All putative linked records where a variable differed, as well as all putative non-linked records, were manually reviewed by Tongan health staff familiar with the reporting systems and naming conventions in Tonga. Final lists of reconciled unique births and deaths were then produced.

#### Capture-recapture analysis

The completeness of the reconciled dataset in each three-year period was assessed by capture-recapture analyses, which compares proportions of records reported in common across different sources to estimate the number of unreported deaths [27]. The capture-recapture method has been used previously for Tongan mortality data [9], and is less sensitive than indirect methods (e.g. Brass growth balance) to significant population movement. The annual emigration rate in Tonga was 1.9% over 2006–2011 [28]. The age groups < 5 years, 5–59 years, and > 60 years were analysed separately, to account for age variation in death rates. Capture-recapture analyses require data sources with the same coverage of the population to be investigated. Hospital discharge records are from Tonga’s main hospital only, whereas the MCCD, community nursing reports and civil registry each covers the entire population. Consequently, to meet the population coverage assumption of the sources, the capture-recapture analysis included three-source categories: (1) MCCDs and hospital discharge records combined, (2) civil registry, and (3) RHS community nursing reports. Two-source analysis relies on the assumption that the sources are independent, whereas three-source analysis can account for dependencies between sources (where reporting a death to one source influences the probability that the death will be reported to another source).

The three-source model included dependence between the MCCD (combined with hospital discharge) and RHS, and between the MCCD (combined with hospital discharge) and the civil registry, as previously performed by Hufanga et al. [9] although without the hospital discharge records. There is a positive dependence between the MCCD database and RHS nursing reports because the MoH manages both sources, and the same staff complete the RHS nursing reports and the notice of death forms that doctors use to complete the MCCD for deaths in the community setting. There is a positive dependence between the MCCD database and the civil registry because the civil registry generally requests a MCCD, if available, for death registration. The selected three-source model was also based on the statistical significance of associations between sources (maximum likelihood) and goodness-of-fit criteria (Bayesian Information Criterion and the Akaike Information Criterion). The dependencies were incorporated using log-linear modelling that included three parameters, one for each source, plus two interaction-terms for the pair-wise dependencies. The GENMOD procedure in SAS 9.4 was used for this analysis.

#### Mortality statistical analysis

The reconciled reported deaths for 2010–2018 were aggregated into triennia (2010–2012, 2013–2015, and 2016–2018) to reduce stochastic fluctuations in annual mortality rates due to small numbers. The mortality data were tabulated by sex, and by age group: < 12 months, 1–4 years, five-year age intervals from 5 years up to 74 years, and > 75 years. Age-specific mortality rates were calculated using total live births as the denominator for age groups < 1 year and < 5 years, and Census population estimates within ages 1–4 years, and for each age group 5 years and over. Denominator populations by age and sex for the triennial mortality rates were calculated by applying linear interpolation or projection to the 2006, 2011 and 2016 Census populations [15, 29, 30]. Confidence intervals (95%CI) for mortality rates are based on the Poisson method for < 100 deaths [31], or normal approximation of the binomial for ≥ 100 deaths [32]. Logarithmic graphs of the triennial age-specific rates have been provided as additional files (see Additional Files: Fig. S1 and Fig. S2.).

Life tables were constructed for each sex and triennium based on the age-specific mortality rates [33]. Confidence intervals for LE were calculated based on the Chiang method [34]. Infant mortality rates (IMR) (probability of dying between birth and age 1 year, _1_q_0_), and U5MR (probability of dying between birth and age 5 years, _5_q_0_), were calculated based on the death rates, with total live births as the denominator, using the life table method, with variances estimated using the Chiang method [34]. Measures of adult mortality were calculated as cumulative probabilities (reported as percentage %) of dying over ages 15–59 years (_45_q_15_), ages 15–64 years (_50_q_15_), ages 15–34 years (_20_q_15_), and ages 35–59 years (_25_q_35_), derived from adult age-specific death rates [33]. All life tables and mortality calculations were performed using the Life Table and Probability of Dying Calculation Tool developed by the authors (RT, SM) [35]. Statistical significance of secular changes over the triennia by sex were assessed by Poisson regression of age-specific counts of deaths (offset by the log of the denominator population) against each triennia (*p* < 0.05) using GENMOD procedure in SAS 9.4, and by examining 95%CIs.

### Comparison of 2010–2018 empirical Tonga mortality data with available published estimates

Available published mortality estimates for Tonga were obtained from Tongan Government websites (MoH and Department of Statistics), and relevant international agencies. National sources include the MoH annual report [36], Tonga Census reports [22, 28], Tonga 2019 Multiple Indicator Cluster Survey (MICS) [37], and Tonga 2012 Demographic and Health Survey (DHS) [38]. Modelled mortality estimates by international agencies include: the United Nations Inter-agency Group for Child Mortality Estimation (UN-IGME) [39]; WHO Global Health Estimates [40]; the Global Burden of Disease (GBD) Study by the Institute of Health Metrics and Evaluation [41]; the World Bank [42]; and the United Nations Statistical Yearbook [43].

The estimates from the external sources were examined for their plausibility and consistency with the empirical estimates. Confidence intervals were not available for estimates from the Tongan MoH report, Tongan 2011 and 2016 Censuses, Tongan 2019 MICS, Tongan 2012 DHS by sex, WHO Global Health Estimates, World Bank, and United Nations Statistical Yearbook. The UN-IGME and GBD report Bayesian uncertainty intervals (UI) with their estimates to account for errors. Biases associated with data inputs, uncertainties in the model parameters, and methodological differences make UI incomparable with frequentist confidence intervals (CI). The UN-IGME provides 90%UIs for the child mortality estimation, which is not comparable with the more conventional wider 95%UI. Direct comparison between 90%UI and 95%UI, and frequentist 95%CI of estimates are therefore not valid. Given differences in data inputs and estimation methods, the published estimates were plotted with the empirical estimates over 2010–2018 for comparison purposes and were not included in the statistical analyses of mortality levels and trends. Estimates for the same year were slightly offset in the graph for display purposes. Details for each source estimate are in the graph footnotes.

#### Ethics approval

This study was approved by the UNSW Human Research Ethics Committee (HC180791) and the Tonga National Health and Research Ethics Committee (120718).

## Results

### Analyses of 2010–2018 Tonga mortality data

#### Total reported deaths and reporting completeness

2274 unique deaths were recorded in 2010–2012; 2503 deaths in 2013–2015; and 2501 deaths in 2016–2018 (Table 1). Over 2010–2018, 63–77% of total reconciled deaths had a MCCD, 55–72% were recorded in community nursing reports, and 50–69% in the civil registry (Table 1).

**Table 1:** Reported deaths by source, Tonga, 2010-2018^a^

Year	Civil registry		MCCD		Hospital discharge^b^		Community nursing reports		Reconciled deaths ^d^
	N	% ^**c**^	N	% ^**c**^	N	% ^**c**^	N	% ^**c**^	
2010	409	55.1	524	70.6	121	16.3	410	55.3	742
2011	387	50.1	597	77.3	143	18.5	469	60.8	772
2012	389	51.2	566	74.5	163	21.4	478	62.9	760
2010–12	**1185**	52.1	**1687**	74.2	**427**	18.8	**1357**	59.7	**2274**
2013	462	60.9	573	75.5	158	20.8	516^e^	68.1	758^f^
2014	596	63.5	722	76.9	188	20.0	660	70.3	940
2015	531	66.0	598	74.3	136	16.9	572	71.1	805
2013–15	**1589**	**63.5**	**1893**	75.6	**482**	19.3	**1748**	69.9	**2503**
2016	546	65.8	645	77.7	125	15.1	578	69.7	829
2017	576	68.9	530	63.4	132	15.8	592	70.7	837
2018	562	67.3	597	71.5	104	12.5	601	72.0	835
2016–18	**1684**	**67.3**	**1772**	70.9	**361**	14.4	**1771**	70.8	**2501**

Abbreviations: *MCCD* medical certificate of cause of death, *N* Number of deaths recorded in the source

^a^Excludes overseas deaths, and deaths among non-residents and foreign nationals

^b^Combined with MCCD in the three-source capture-recapture analysis to assess reporting completeness

^c^Reporting completeness of the source as a percentage of the reconciled deaths

^d^Unique deaths from all four sources deduplicated and reconciled using deterministic and probabilistic linkage

^e^This figure is incomplete as the community nursing reports for six health centres in Tongatapu and the health centres in Ha’apai and Vava’u were not available.

^f^This figure is likely to be an undercount of the number of deaths in 2013 due to unavailable community nursing reports

The estimated reporting completeness of the reconciled deaths for each period was: 98.4% (95%CI: 97.4–99.0%) for 2010–2012; 98.1% (95%CI: 97.4–98.5%) for 2013–2015; and 97.5% (95%CI: 96.7–98.1%) for 2016–2018. There was minimal variation in the reporting completeness across age groups. Given that the estimated completeness of the reconciled death data exceeded 95%, the number of total deaths and observed mortality measures were not adjusted.

#### Infant and under-five mortality

**Fig. 2:**
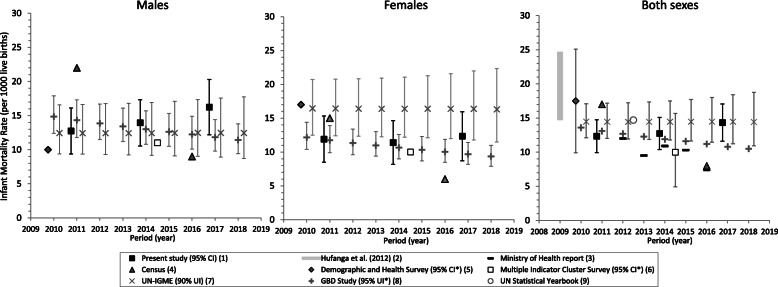
Estimates of infant mortality rate (IMR) by sex and data source^a^, Tonga, 2010–2018. ^a^ Estimates by international agencies and previous published reports are presented with present study empirical estimates for comparison purposes only and are not included in trend analysis. ^(1)^ Estimates from the present study based on reconciled routinely collected data for 2010–2012, 2013–2015, and 2016–2018 are shown with 95% confidence intervals (CI) at years 2010.75, 2013.75, and 2016.75 respectively. ^(2)^ Source: [9]. Range of estimates for 2005–2009 based on reconciled data is shown at year 2009. The lower and upper limits of the range represent estimates adjusted by two-source and three-source capture-recapture analysis. ^(3)^ Source: 2016 Annual Report based on Reproductive Health Services community nursing reports [36] ^(4)^ Source: 2011 Census [28] and 2016 Census [22]. ^(5)^ Source: 2012 Survey [38]. IMR by sex refers to the 10-year period preceding the survey in 2012, confidence intervals are not available. IMR (both sexes) refers to the 5-year period preceding 2012 with 95%CI*. Estimates shown offset at year “2009.75”. ^(6)^ Source: 2019 Survey [37]. IMR calculated for the 10-year period preceding the survey in 2019. 95%CI are not available for IMR by sex*. Estimates shown offset at year “2014.5”. ^(7)^ Source: United Nations Inter-agency Group for Child Mortality Estimation [39]. IMR derived from under-five mortality using model life table (West). Sex-specific IMR estimates based on B-splines regression model. Annual estimates over 2010–2018 are shown offset at + 0.25 per year. 90% uncertainty intervals (UI) are not comparable to CI. ^(8)^ Source: [41]. Estimates modelled from spatiotemporal and Gaussian process regressions. *95%UI are available by sex only. 95%UI are not comparable to 95%UI and CI. ^(9)^ Source: 2019 Edition [43]. Estimate shown at year 2012.5 is an average over 2010–2015

**Fig. 3:**
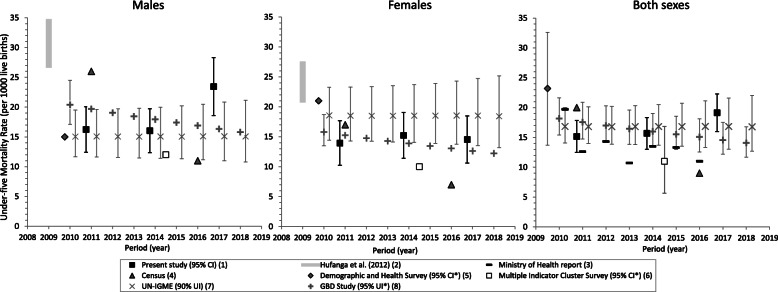
Estimates of under-five mortality rate (U5MR) by sex and data source^a^, Tonga, 2010–2018. ^a^ Estimates by international agencies and previous published reports are presented with present study empirical estimates for comparison purposes only and are not included in trend analysis. ^(1)^ Estimates from the present study based on reconciled routinely collected data for 2010–2012, 2013–2015, and 2016–2018 are shown with 95% confidence intervals at years “2010.75”, “2013.75”, and “2016.75” respectively. ^(2)^ Source: [9] Range of estimates for 2005–2009 based on reconciled data is shown at year 2009. The lower and upper limits of the range represent estimates adjusted by two-source and three-source capture-recapture analysis. ^(3)^ Source: 2016 Annual Report [36], based on Reproductive Health Services community nursing reports. ^(4)^ Source: 2011 Census [28] and 2016 Census [22]. 2011 estimate (both sexes) is shown offset at year “2010.75”. ^(5)^ Source: 2012 Survey [38]. U5MR by sex refers to the 10-year period preceding the survey in 2012, confidence intervals are not available. U5MR (both sexes) refers to the five-year period preceding 2012 with 95% confidence interval. Estimates are shown offset at year “2009.75”. ^(6)^ Source: 2019 Survey [37]. U5MR calculated for the 10-year period preceding the survey in 2019. 95%CI are not available for U5MR by sex*. Estimates shown offset at year “2014.5”. ^(7)^ Source: United Nations Inter-agency Group for Child Mortality Estimation [39] Sex-specific U5MR estimates based on B-splines regression (BSR) model. Total U5MR estimate based on Bayesian B-splines bias-adjusted model. Annual estimates over 2010–2018 are shown with 90% uncertainty intervals offset at + 0.25 per year. 90% uncertainty intervals (UI) are not comparable to 95%UI and CI^(8)^ Source: [41]. Estimates modelled from spatiotemporal and Gaussian process regressions. *95% uncertainty intervals not available by sex for years 2011–2018. 95%UI are not comparable to 95%CI

Between 2010–2012 and 2016–2018, the estimated IMR (Fig. 2) varied between 12 and 14 per 1000 live births (95%CI: 10–17), and U5MR (Fig. 3) 15–19 per 1000 live births (95%CI: 12–22). Male U5MR increased from 16 to 23 per 1000 live births (*p* = 0.0165), significantly higher than females at 2016–2018 (*p* = 0.0033) (Table 2, Fig. 3). IMR by sex did not change significantly: 13–16 per 1000 live births (95% CI: 9–20) for males and 11–12 (95%CI: 8–16) for females (Table 2, Fig. 2).

**Table 2:** Summary mortality estimates in Tonga, 2010–2018, by sex, from empirical data

Indicator	Males			Females		
	2010-12^**a**^	2013–15	2016–18	2010-12^**a**^	2013–15	2016–18
Infant mortality rate per 1000	12.7 (9.4–16.1)	13.9 (10.5–17.3)	16.3 (12.2–20.3)	11.9 (8.5–15.3)	11.3 (8.1–14.6)	12.3 (8.7–16.0)
Under-5 mortality rate per 1000^	16.2 (12.4–20.1)	16.0 (12.4–19.7)	23.4^‡^ (18.6–28.3)	14.0 (10.2–17.7)	15.2 (11.4–19.1)	14.6 (10.6–18.5)
Adult mortality (%)^b^^						
15–34 years	3.3 (2.6–4.1)	3.9 (3.1–4.8)	4.5^‡^ (3.6–5.5)	1.3 (0.9–1.9)	1.9 (1.4–2.6)	1.9 (1.4–2.6)
35–59 years	24.9 (22.5–27.3)	25.8 (23.4–28.1)	25.4 (23.1–27.6)	17.4 (15.3–19.4)	17.7 (15.7–19.8)	19.7 (17.6–21.8)
15–59 years	27.4 (25.0–29.7)	28.7 (26.4–31.0)	28.7 (26.4–31.0)	18.5 (16.4–20.5)	19.3 (17.2–21.4)	21.2^†‡^ (19.1–23.3)
15–64 years	37.3 (34.5–39.9)	39.6 (36.9–42.2)	38.8 (36.2–41.3)	26.4 (23.9–28.9)	27.7 (25.2–30.1)	28.3^‡^ (25.9–30.7)
Life expectancy at birth (years)	65.3 (64.6–66.0)	64.6 (63.9–65.3)	64.4 (63.6–65.1)	69.5 (68.8–70.2)	68.8 (68.1–69.4)	68.8 (68.1–69.5)

95% confidence intervals shown in brackets

^a^Denominators for the infant mortality and under-five mortality rates for 2010–2012 are live births data collected by Ministry of Health only. Civil registry birth data were not available for the 2010–2012 period

^b^probability of dying between the ages indicated, inclusive

^ Difference between sexes significant (*p* < 0.05)

†Test for trend over 2010–2018 significant (*p <* 0.05)

‡Difference between 2010 and 2012 and 2016–2018 significant (*p <* 0.05)

#### Adult mortality

**Fig. 4:**
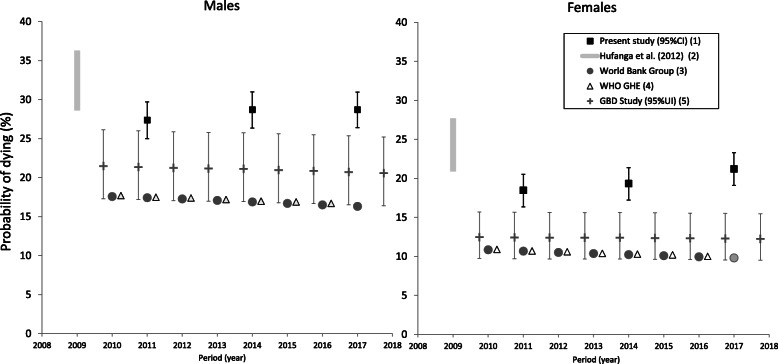
Estimates of adult mortality rate (15–59 years) by sex and data source^a^, Tonga, 2010–2018. ^a^ Estimates by international agencies and previous published reports are presented with present study empirical estimates for comparison purposes only and are not included in trend analysis. ^(1)^ Estimates from the present study based on reconciled routinely collected data for 2010–2012, 2013–2015, and 2016–2018 are shown with 95% confidence intervals at years 2011, 2014, and 2017 respectively. ^(2)^ Source: [9] Range of estimates for 2005–2009 based on reconciled data is shown at year 2009. The lower and upper limits of the range represent estimates adjusted by two-source and three-source capture-recapture analysis. ^(3)^ Source: [42]. ^(4)^ Source: WHO Global Health Estimate [40]. Annual estimates over 2010–2017 are shown offset at + 0.25 per year. ^(5)^ Source: [41]. Estimates modelled from spatiotemporal and Gaussian process regressions. Annual estimates over 2010–2018 are shown with 95% uncertainty intervals offset at − 0.25 per year. 95%UI are not comparable to 95%CI

Over 2010–2018, cumulated risks of adult mortality (< 65 years) were significantly higher in men than women (non-overlapping 95%CIs) (Table 2). For 15–34 years, estimated male cumulated mortality was 3–5%, approximately twice that in women. For 15–59 years, cumulated mortality in men was 27–29%, while in women, it increased from 18% in 2010–2012 to 21% in 2016–2018 (trend, *p* = 0.04) (Table 2, Fig. 4). For 15–64 years, cumulated mortality in men over 2010–2018 was 37–39%, and in women 26–28%. Cumulated mortality in both sexes over the period was mostly concentrated in age group 35–59 years (Table 2).

#### Life expectancy

**Fig. 5:**
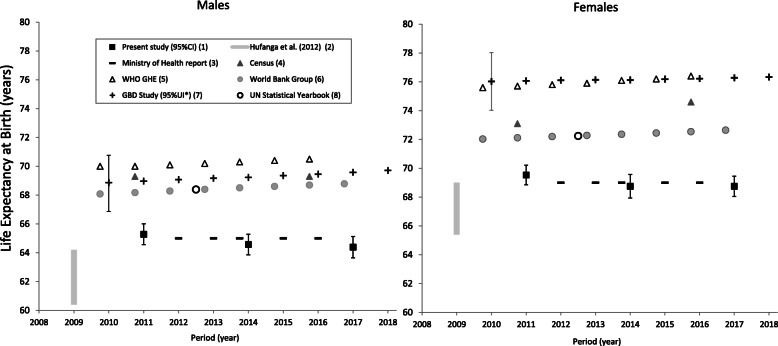
Estimates of life expectancy (LE) at birth by sex and data source^a^, Tonga, 2010–2018. ^a^ Estimates by international agencies and previous published reports are presented with present study empirical estimates for comparison purposes only and are not included in trend analysis. ^(1)^ Estimates from the present study based on reconciled routinely collected data for 2010–2012, 2013–2015, and 2016–2018 are shown with 95% confidence intervals at years “2010.75”, “2013.75”, and “2016.75” respectively. ^(2)^ Source: [9] Range of estimates for 2005–2009 based on reconciled data is shown at year 2009. The lower and upper limits of the range represent estimates adjusted by two-source and three-source capture-recapture analysis. ^(3)^ Source: 2016 Annual Report [36]. ^(4)^ Source: 2011 Census [28] and 2016 Census [22]. Estimates are shown offset at years “2010.75” and “2015.75”. ^(5)^ Source: WHO Global Health Estimate [40]. ^(6)^ Source: [42]. Estimates over 2010–2017 are shown offset at − 0.25 for each year. ^(7)^ Source: [41]. Estimates modelled from spatiotemporal and Gaussian process regressions. *95% uncertainty intervals not available by sex for years 2011–2016. 95%UI are not comparable to 95%CI. ^(8)^ Source: 2019 Edition [44]. Estimate shown at year 2012.5 is an average over 2010–2015

LE at birth was estimated at 64–65 years for men, and 69–70 years for women for 2010–2012 and 2013–2018 (Table 2, Fig. 5).

### Comparisons of empirical Tonga mortality data analyses and international agencies and other estimates

Mortality estimates reported by country sources (MoH report, Censuses, MICS, and DHS), and modelled by international agencies are presented with the empirical study estimates over 2010–2018 in Figs. 2, 3, 4 and 5. The MoH reported IMR (7 per 1000 births) and U5MR (11 per 1000 births) in 2016 [36] that were markedly lower than the corresponding empirical estimates of the present study (Figs. 2-3). The MoH annual LE estimates remained constant over 2012–2016 at 65 years for men and 69 years for women, which were consistent with the empirical estimates of this study (Fig. 5).

From the 2011 Tongan Census, estimates of male IMR and U5MR were 1.6 times higher than the corresponding 2010–2012 male empirical estimates, but female IMR and U5MR were comparable to the 2010–2012 female empirical estimates (Figs. 2-3). In contrast, estimates of IMR (male: 9, female: 6 per 1000 births) and U5MR (male: 11, female: 7 per 1000 births) from the 2016 Census calculations were lower than all reported estimates and the 2016–2018 empirical figures. The 2011 and 2016 Census estimates of LE at birth were 3 years higher for males and 1–3 years higher for females than the equivalent 2010–2012 and 2016–2018 empirical estimates from the present study (Fig. 5).

The MICS estimates of IMR (male: 11, female: 10 per 1000 births) and U5MR (male: 12, female: 10 per 1000 births) for 2008–2018 fell within the lower range of the present empirical estimates (Figs. 2-3); the 95%CI for total IMR was 5–16 per 1000 births and for total U5MR 6–17 per 1000 births. The DHS estimate of male IMR (10 per 1000 births) and U5MR (15 per 1000 births) for 2002–2011 also fell within the lower range of the present empirical male estimates, however, the DHS estimates of female IMR (17 per 1000 births) and U5MR (21 per 1000 births) were ~ 1.5 times those of the empirical female estimates (Figs. 2-3). The DHS 95%CI for sex-specific child mortality were not available, however, total IMR (10–25 per 1000 births), and total U5MR (14–33 per 1000 births) for 2007–2011 are in Figs. 2-3; the estimates from the other sources fell within the lower limits of these ranges.

International modelled estimates of IMR and U5MR for males were broadly consistent with the empirical estimates of the present study for males, but the UN-IGME estimates for females were considerably higher (~ 30%) than the UN-IGME estimates for males, and those reported for females in this empirical study (Figs. 2-3).

International modelled estimates of cumulated adult mortality (15–59 years) risk were consistently and markedly lower than the empirical estimates in the present study over 2010–2018; in men they were 17–18% (WHO) and 21% (95%UI: 16–25%) (GBD); and in women they were 10–11% (WHO) and 12% (95%UI: 10–16%) (GBD) (Fig. 4). Corresponding estimates of LE at birth throughout 2010–2018 in males were 4–7 years higher than the empirical LE estimates in this study, at 68–70 years (GBD, World Bank), and 70–71 years (WHO); and in females the modelled estimates were 3–6 years higher over the period at 72 years (UN, World Bank) and 76 years (GBD, WHO) (Fig. 5).

## Discussion

Based on reconciled empirical data estimated as > 95% complete, the present study provides evidence for considerable adult mortality over three triennia in 2010–2018 which is limiting improvements in LE in Tonga, despite relatively low levels of infant and child mortality. Male U5MR increased between 2013–2015 and 2016–2018 due to a number of coincidental deaths in ages 1–4 years in 2016 (*n* = 14) which local health staff verified; half of these deaths were from external causes (predominantly motor vehicle accidents). Levels of premature adult mortality during 2010–2018 were significantly higher in men than in women, although mortality in women increased. LE at birth remained at 64–65 years for men, and 69–70 years for women over the period. These are levels at which LE gains would be expected, as exemplified in countries presently with relatively high LE; historical gains of + 1–2 years per 5-year intervals have occurred in the context of reduced population health risk factors and improved social and economic conditions [45, 46].

As countries move through the epidemiological transition, increases in adult mortality from non-communicable diseases (NCDs), particularly major cardiovascular diseases (CVD), diabetes-related conditions and injuries, may counteract the effects of declines in early child mortality, resulting in stagnation in LE. This occurred in Australia during 1945–1970 for males, and 1960–1970 for females [47, 48], before subsequent declines in premature cardiovascular mortality in adults restored historical trend increases in LE. The indication of considerable premature adult mortality in Tonga is comparable to the experience of other Pacific Island countries, such as Fiji [11, 12], Nauru [49], Vanuatu [50], Kiribati [13], and the Federated States of Micronesia [51]. The empirical levels of adult mortality in Tonga are also reflected in the age-specific pattern of mortality derived from the reconciled deaths (see Supplementary Fig. S1 and Fig. S2).

The level of premature adult mortality is consistent with the significant NCD illness burden reported in Tonga, especially from atherosclerotic CVD and type 2 diabetes (T2DM) and their risk factors [52, 53]. Using corrected cross-sectional population surveys among adults (25–64 years) in Tonga, Lin et al. [52] projected increasing T2DM prevalence over 2012–2020 from 15 to 20% in men, and from 20 to 24% in women. These projected period trends were driven by rising obesity prevalence (~ 3% increase every 5 years in men and 1.3% increases in women), which Lin et al. [52] projected to be 65% (men) and 81% (women). For other CVD risk factors, the 2012 cross-sectional STEPwise approach to surveillance (STEPS) survey among 25–64 years found that the prevalence of hypertension was ~ 28% and raised blood cholesterol was ~ 49% (both sexes). At ages > 45 years, hypertension prevalence was higher in women (53%) than men (41%), and elevated raised blood cholesterol was higher in women (68%) than men (60%) [53]. Tobacco use was 46% in men, and 13% in women [53]. The Tonga 2017 STEPS survey among 18–69 years found prevalence of obesity as 67% in men, 83% in women; hypertension as 36% in men, 38% in women; raised blood cholesterol as 32% in men, 34% in women; and tobacco use as 40% in men, and 16% in women [54]. In the present study, the bulk of adult mortality over 2010–2018 occurred at ages 35–59 years. Young adult (15–34 years) deaths are more likely from injuries such as transport accidents. Detailed analysis of causes of death by age and sex, and life table decomposition by age and cause-specific contributions, are required to further understand the observed mortality and LE levels.

Previous empirical assessment of Tongan mortality for 2001–2004 and 2005–2009 was based on less than complete datasets, and limited data from the outer islands [9]. Datasets for 2001–2004 were more limited than for 2005–2009. Based on reconciled deaths, the previous completeness of the civil registry was estimated at 55%, medical certificates 67%, and community nursing datasets 35% [9], which is much lower than in the present study. Capture-recapture analysis of the reconciled data for 2005–2009 estimated completeness as 58–88% [9], also considerably lower than the > 95% estimated completeness of the reconciled data for 2010–2018 used in the present study, which included hospital discharge data as well. Although the mortality estimates calculated by Hufanga et al. [9] were adjusted for under-reporting, these were based on the estimated completeness of data for the main island (Tongatapu) only. These analyses produced adjusted mortality that were generally higher, and LE estimates that were lower, than those found here (Figs. 2, 3, 4 and 5). Given the uncertainty around the level of completeness of the reconciled data for 2001–2009, it is possible that the extent of unreported deaths for adjusting overall mortality estimation in previous analyses were over-estimated, and thus the mortality measures were also over-estimated. This limits the assessment of mortality trends between the previous study estimates for 2005–2009 and the triennial estimates for 2010–2018 (present study). Nevertheless, the general pattern of considerable premature adult mortality limiting LE improvements is evident.

The IMR and U5MR for 2012–2016 reported in the MoH report [36] were lower than the present study empirical estimates (Figs. 2-3). The MoH estimates are calculated from datasets which were not reconciled and consequently not as complete as those used in the present study. The numerators for the MoH-reported IMR and U5MR were based on RHS community nursing reports only. However, the reported annual LE estimates were consistent with the present study estimates, and correspond with estimates derived from unadjusted reconciled data for 2005–2009 [9].

Census estimates of early child mortality are calculated indirectly using data from Census questions on children ever-born and children surviving (CEBCS) by age group of mother. The large difference in U5MR between the 2011 Census, and the 2016 Census, and the markedly low 2016 Census estimates for U5MR, indicate possible biases from retrospective reporting by mothers of births and surviving children. For mortality Census analyses, the CEBCS data were applied to model life tables to derive the LE estimates: in the 2011 Census, the UN Far East Asian pattern was used for Tongan men and the Coale–Demeny West model for Tongan women [28]; in the 2016 Census, the UN Far East Asian pattern was used for both sexes [22]. However, such model life tables, using child mortality data alone, do not account for the impact of the considerable premature adult mortality on adult age-specific death rates, and therefore are likely to under-estimate adult mortality and over-estimate LE [9, 10]. This may account for the Tongan Census estimates of LE at birth being higher than the empirical estamates of the present study. Based on the life tables generated from the Census data, the number of deaths in 2011 was 699 [28] and in 2016 was 582 [22]; these figures are lower than the empirically-derived reconciled numbers from the present study of 772 and 829 deaths in these years, respectively.

The Tongan household survey estimates of early child mortality also demonstrated some variation. The Tongan MICS (2019) estimates of IMR and U5MR for 2008–2018 were within the lower limit of the empirical estimates. The Tongan DHS (2012) DHS estimate of male IMR and U5MR also fell within the lower range of the male estimates from this empirical study, however, the reported female IMR and U5MR for 2002–2011 were 1.4–1.7 times the reported estimates in males, and ~ 1.5 times those of the empirical female estimates in this study. Estimated IMR and U5MR is expected to be higher in males than females based on recorded data, because male newborns have a greater biological susceptibility to congenital abnormalities, perinatal conditions and infectious diseases than females [55]. IMR estimates that produce higher female IMRs lack construct validity. The DHS and MICS estimates were derived from retrospective full birth history by interviews from samples of Tongan women aged 15–49 years: the DHS included 3068 women (97% response rate) and 1749 under-five children, of which < 95% had their births registered or had a birth certificate [38]; the MICS included 2903 women (92% response rate) and 1347 under-five children which had 98% birth registration [37]. The reported estimates are subject to recall inaccuracy, under-reporting of early infant deaths, and misreporting of age at death [38]. This may explain the sex-specific differences between the DHS (2012) and the empirical estimates**.**

Mortality indices modelled and published by international agencies are based on limited empirical mortality data from Tonga. The WHO [40], GBD [41] and UN-IGME mortality databases [39] published in 2018–2020 include death registration data for years prior to 2006 only, and Census estimates up to 2006; the 2012 DHS was also included in the GBD and UN-IGME databases, and the 2019 MICS preliminary results included by UN-IGME. Differences among agency estimates reflect differences in inclusion criteria and modelling. Like the UN Statistical Yearbook [43] and World Bank [42], the WHO adopts the UN Population Division World Population Prospects life table system [44, 56], which differs from the GBD model life table system [41]. The UN-IGME uses Bayesian B-spline bias-adjusted models for estimating total IMR and U5MR, and B-spline regression models for sex-specific estimates [39] based on a wide range of populations; the higher IMR and U5MR estimates for females than males likely reflects the exclusive use of 2012 Tongan DHS data in the UN-IGME models (see Figs. 2 and 3). The GBD uses spatio-temporal and Gaussian process regressions to model under-5 and adult mortality [41]. Consequently, the models produce smoothed annual mortality rates without the annual fluctuations expected from small population sizes such as Tonga.

The present study has several limitations. Town and District Officers data held by the Ministry of Internal Affairs were not available. Exclusion of these data, however, is likely to have minimal impact as the deaths recorded by Town Officers largely depend on death notifications completed by public health nurses who also complete the community nursing reports (Fig. 1). It is therefore reasonable to expect that most of the subset of deaths recorded by Town and District Officers has been captured in the reconciled data in this study. Community nursing reports varied in quality, with some variables incomplete or missing, and data prior to 2010 are unavailable for assessing longer term trends. In addition, errors in the reconciliation of data sources are considered minor since duplicates and linked records were manually reviewed. Data on Tongans who seek medical care abroad before dying abroad are poorly recorded within reporting systems, but are considered to represent an extremely small number of total deaths. Of the total recorded deaths in 2010–2018 available for this study, 3% died overseas. The potential underestimation of the mortality measures due to non-inclusion of Tongan deaths overseas after medical referral would therefore be minimal.

Despite these limitations, the present study provides mortality estimates based on data from the principal mortality data collections in Tonga that have been reviewed for content errors prior to reconciliation and assessed for reporting completeness. Reconciled mortality data were > 95% complete, although only 74% of the total reported deaths had a MCCD; of these, 93% had a cause of death assigned according to International Classification Diseases 10th revision (ICD-10), and the remaining 7% coded to an ill-defined condition. Key recommendations for strengthening reporting processes and completeness of medical certification and death registration in Tonga and other small countries with fragmentary mortality data include: improvement in cross-sector data access and sharing between civil registries, health departments, national statistics offices and other relevant agencies; strengthening and integration of the reporting of deaths by community health nurses, with a medical certification process; implementation of ongoing training for data management staff; and, establishment procedures for the regular review and quality assurance of death records within each reporting agency, including routine reconciliation of death data from all sources.

## Conclusion

Over the past decade, the availability and completeness of data sources for analysing mortality levels in Tonga have increased considerably. This study provides evidence of plateaux in LE based on updated empirical linked records, with higher premature adult mortality and lower LE than indirect and modelled estimates for the same period based on limited and incomplete data sources. These findings highlight the importance of ensuring that official mortality estimates for health monitoring and planning are derived from empirical data appropriately assessed for coverage and completeness.

## Supplementary Information

**Additional file 1: Fig. S1.** Logarithmic graph of male age-specific death rates per 1000, Tonga, by triennia, 2010–2018

**Additional file 2: Fig. S2.** Logarithmic graph of female age-specific death rates per 1000, Tonga, by triennia, 2010–2018

## Data Availability

Empirical datasets analysed during the current study are not publicly available due to ethical restriction (contains personal identifiable information) and are held on secure servers with restricted access. Access was obtained through the approval of the research ethics committee and data custodians. Datasets from international agencies supporting the conclusions of this article are publicly available in the World Health Organization Global Health Observatory data repository, https://apps.who.int/gho/data/node.main.686?lang=en [57], Global Burden of Disease study Global Health Data Exchange http://ghdx.healthdata.org/gbd-2019 [41], and United Nations Inter-agency Group for Child Mortality Estimation web portal https://childmortality.org/data [39].

## Abbreviations

CEBCS: Children ever-born and children surviving
CI: Confidence interval
DHS: Demographic and Health Survey
GBD: Global Burden of Disease Study
IMR: Infant mortality rate
LE: Life expectancy
MCCD: Medical certificates of cause of death.
MICS: Multiple Indicator Cluster Survey
MoH: Ministry of Health
NCD: Non-communicable disease
RHS: Reproductive Health Services
SDG: Sustainable Development Goal
U5MR: Under-five mortality rate
UN: United Nations
UN-IGME: United Nations Inter-agency Group for Child Mortality Estimation
UI: Uncertainty interval
WHO: World Health Organization
