# Supplementary figures and images for: Mortality in Tonga over three triennia, 2010–2018
Source: BMC Public Health. 2021 Jan 6;21:36. doi: 10.1186/s12889-020-10023-w (PMC7789386; doi:10.1186/s12889-020-10023-w)

## Slide 1
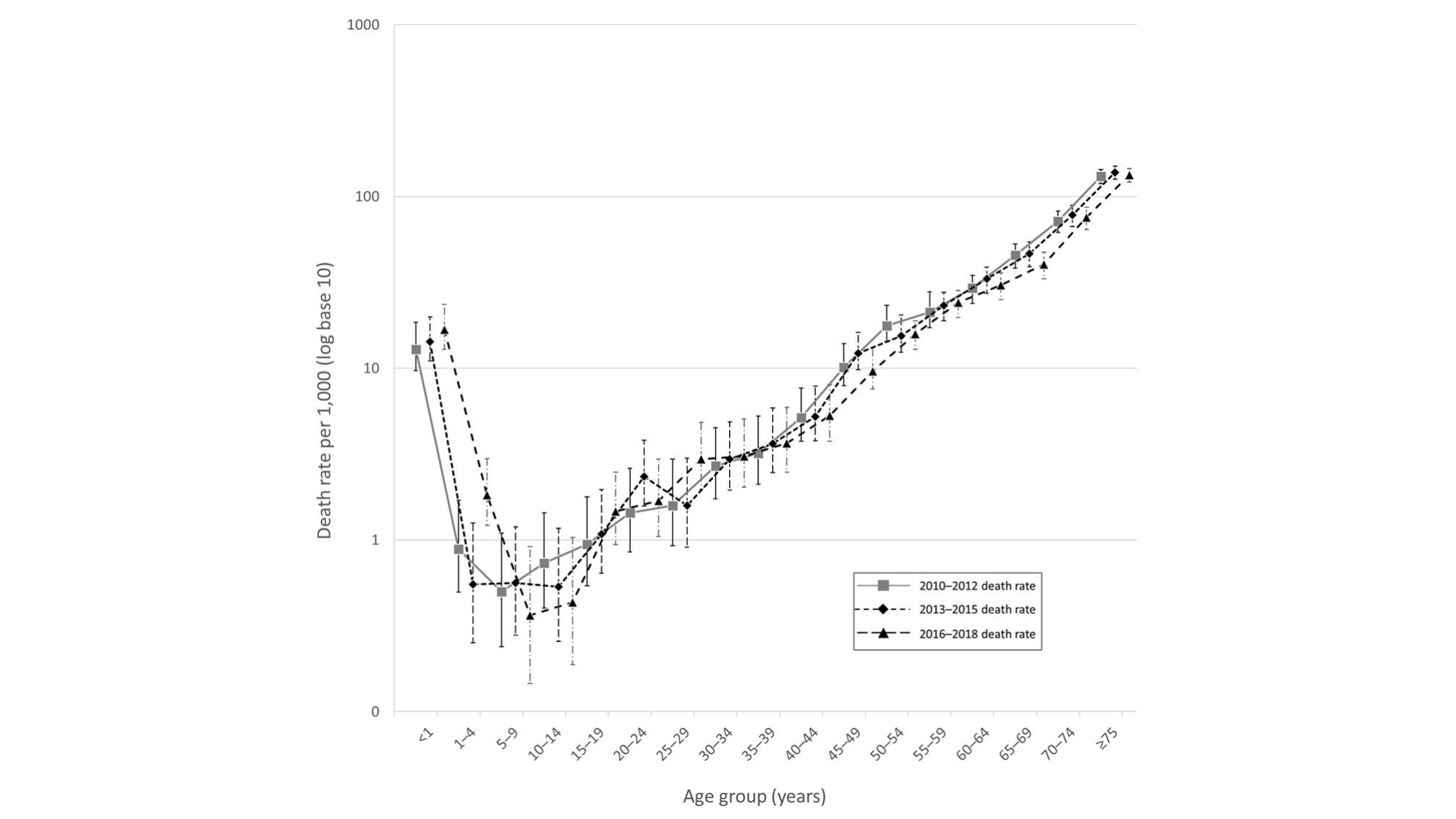

Supplement: Supplementary file 1 — Additional file 1: Fig. S1. Logarithmic graph of male age-specific death rates per 1000, Tonga, by triennia, 2010–2018 [file 12889_2020_10023_MOESM1_ESM.pptx]

## Slide 1
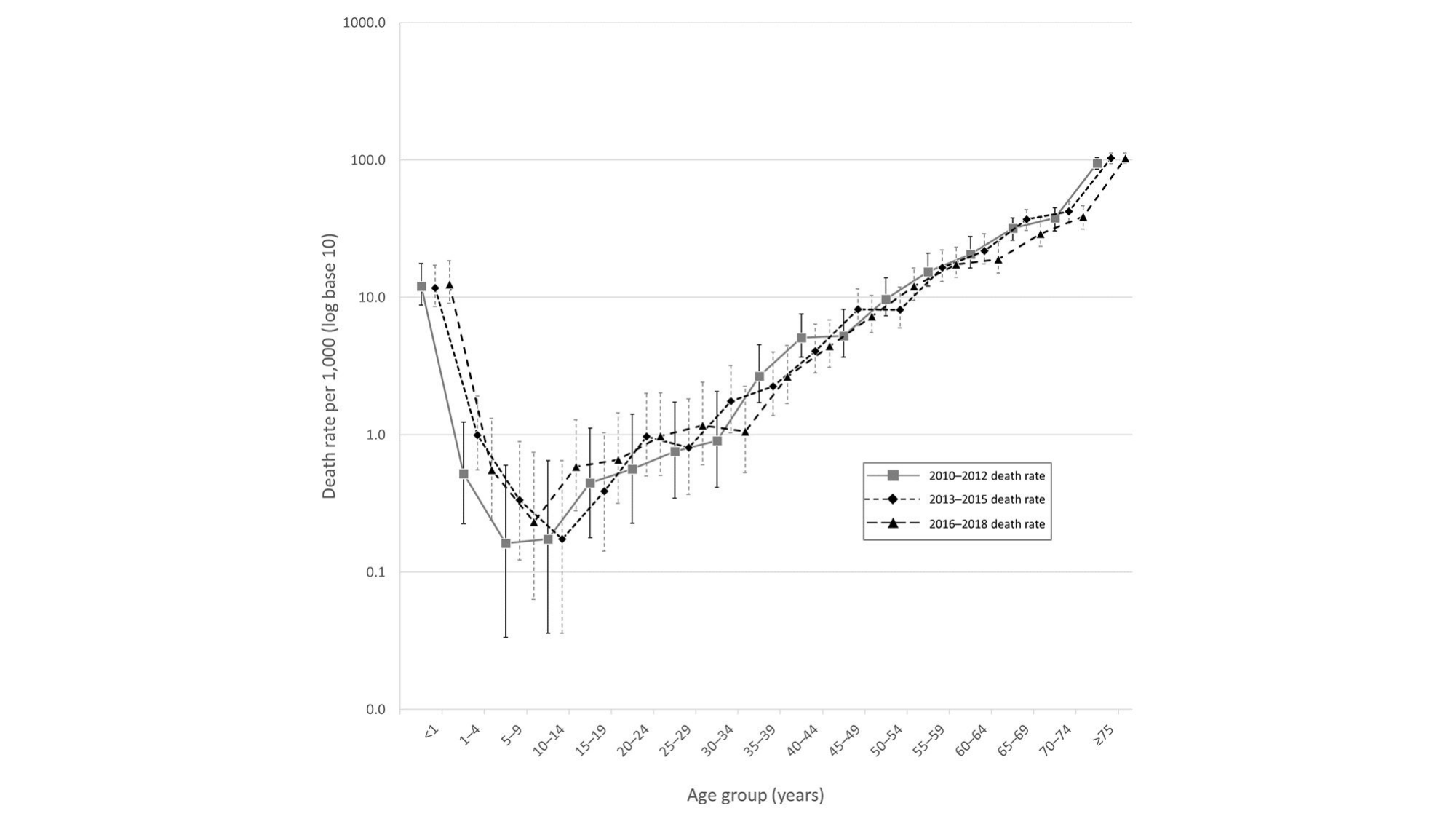

Supplement: Supplementary file 2 — Additional file 2: Fig. S2. Logarithmic graph of female age-specific death rates per 1000, Tonga, by triennia, 2010–2018 [file 12889_2020_10023_MOESM2_ESM.pptx]
